# Information-Based Similarity of Ordinal Pattern Sequences as a Novel Descriptor in Obstructive Sleep Apnea Screening Based on Wearable Photoplethysmography Bracelets

**DOI:** 10.3390/bios12121089

**Published:** 2022-11-28

**Authors:** Mingjing Chen, Shan Wu, Tian Chen, Changhong Wang, Guanzheng Liu

**Affiliations:** 1 School of Biomedical Engineering, Sun Yat-Sen University, Shenzhen 518107, China; 2 Alfred E. Mann Department of Biomedical Engineering, University of Southern California, Los Angeles, CA 90089-1112, USA

**Keywords:** obstructive sleep apnea (OSA), information-based similarity of ordinal pattern sequences (OP_IBS), photoplethysmography (PPG), wearable bracelets

## Abstract

Obstructive sleep apnea (OSA) is a common respiratory disorder associated with autonomic nervous system (ANS) dysfunction, resulting in abnormal heart rate variability (HRV). Capable of acquiring heart rate (HR) information with more convenience, wearable photoplethysmography (PPG) bracelets are proven to be a potential surrogate for electrocardiogram (ECG)-based devices. Meanwhile, bracelet-type PPG has been heavily marketed and widely accepted. This study aims to investigate the algorithm that can identify OSA with wearable devices. The information-based similarity of ordinal pattern sequences (OP_IBS), which is a modified version of the information-based similarity (IBS), has been proposed as a novel index to detect OSA based on wearable PPG signals. A total of 92 PPG recordings (29 normal subjects, 39 mild–moderate OSA subjects and 24 severe OSA subjects) were included in this study. OP_IBS along with classical indices were calculated. For severe OSA detection, the accuracy of OP_IBS was 85.9%, much higher than that of the low-frequency power to high-frequency power ratio (70.7%). The combination of OP_IBS, IBS, CV and LF/HF can achieve 91.3% accuracy, 91.0% sensitivity and 91.5% specificity. The performance of OP_IBS is significantly improved compared with our previous study based on the same database with the IBS method. In the Physionet database, OP_IBS also performed exceptionally well with an accuracy of 91.7%. This research shows that the OP_IBS method can access the HR dynamics of OSA subjects and help diagnose OSA in clinical environments.

## 1. Introduction

Obstructive sleep apnea (OSA) is a common sleep disorder, characterized by recurrent episodes of reduced or absent breathing during sleep [1]. It is estimated that 936 million adults suffer from OSA worldwide [2]. Aside from a lower sleep quality, OSA can also lead to some fatal events, including cardiovascular diseases [3], cerebrovascular diseases and even sudden death [4].

The gold standard method for diagnosing OSA is polysomnography (PSG) [5]. However, it is expensive, bulky, and multichannel, which makes people reluctant to undergo this process and thus leads to around 85% of OSA patients being undiagnosed [2]. Therefore, there is an urgent demand for more convenient and accessible OSA screening tools. Because OSA is associated with autonomic nervous system (ANS) dysfunction, heart rate variability (HRV), being able to assess ANS functioning [6], is believed to be a powerful approach to investigating OSA [7,8]. Numerous recent studies have focused on OSA screening based on single-channel electrocardiogram (ECG) signals [9,10].

Having a high agreement in RR extraction (the time elapsed between two successive R waves of the QRS signal on the ECG), photoplethysmography (PPG) and pulse rate variability (PRV) have shown great potential to be surrogates for ECG-based HRV analysis [11,12]. Previous studies have shown the feasibility of PPG monitoring for sleep apnea. Karmakar et al. argued that the PPG signal reflects respiratory arousals in sleep apnea patients [13]. Gil et al. explored the utility of PPG in OSA screening for children and achieved an accuracy of 86.7% [14]. Bracelet-type PPG has been widely accepted and heavily marketed because of its low cost and high convenience [15]. Compared with ECG patches or belts, it is much easier to wear without hampering daily activities.

With the awareness that linear methods are not suitable for the analysis of nonlinear ANS systems, nonlinear methods are being developed vigorously, such as entropy [10], correlation dimension [16] and empirical mode decomposition [17]. However, most nonlinear methods are only applicable to stable and noiseless signals. Sensitive to motion artefacts and environmental noise, PPG data from off-the-shelf wearables are usually highly interfered and have a low signal-to-noise ratio [15]. Consequently, most nonlinear approaches are ineffective in analyzing such PPG signals.

Ordinal pattern (OP) describes a nonlinear relationship within a short segment according to the order of consecutive values [18]. In addition to detecting possible regularities in the time series, it is also robust against noise [19]. Recently, more researchers have focused on its application in biosignal processing. Frank et al. clarified fetal behavioral states by applying an OP to heart rate variability [20]. Nicolaou et al. analyzed the OP of an epileptic electroencephalogram (EEG) and achieved an accuracy of 86.1% in detection [21]. Capable of assessing the similarity between two-symbol series, the information-based similarity (IBS) index has been proven effective in physiological state monitoring. Cui et al. found that the IBS index was powerful in clarifying atrial fibrillation [22]. Baumert et al. argued a reduced information domain similarity with aging [23]. Our previous study applied the IBS index to ECG signals to detect OSA patients and achieved promising results. However, the traditional IBS method is highly dependent upon the increase or decrease in adjacent RR intervals. This may result in some useful large-scale characteristics being neglected.

In the present study, information-based similarity of ordinal pattern sequences (OP_IBS) is proposed to capture time-series characteristics more comprehensively. The PP (the time elapsed between two successive P waves in the PPG) intervals were transformed into ordinal patterns, and then the similarity among different segments was quantized. We hypothesized that the dynamic change in short-term heart fluctuations can be better reflected by fully considering the relationships within the same episodes.

## 2. Materials and Methods

**Figure 1:**
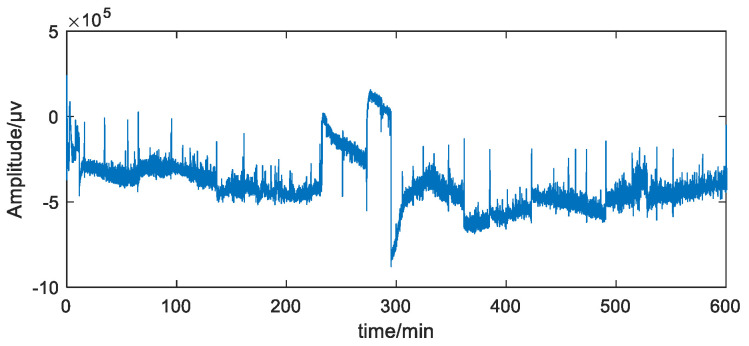
Overnight wearable PPG signal.

**Figure 2:**
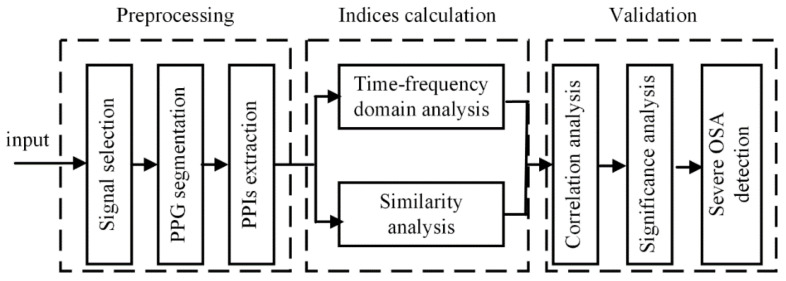
The framework of proposed PRV analysis method.

In this study, PPG signals obtained from commercial bracelets are analyzed, along with simultaneously collected PSG signals as the gold standard [24,25]. The PPG bracelet is an optical device that is often used for pulse rate monitoring based on the detection of blood volume changes in the microvascular bed of tissue. An example diagram of an overnight wearable PPG signal is shown in Figure 1. Overnight PSG was conducted using Compumedics Sleep System (Compu-medics, Melbourne, Australia). The framework of the PRV analysis method is shown in Figure 2. First, PP intervals were extracted and segmented. Then, the OP_IBS index, along with other classical indices, was calculated. Next, a correlation analysis and significance analysis were performed to prove the effectiveness. Finally, the machine learning algorithms were applied to OSA detection tasks.

### 2.1. Data

**Figure 3:**
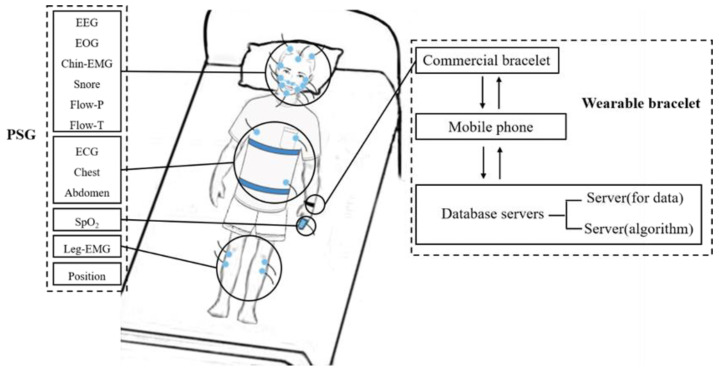
The experimental system diagram.

In this study, the same data as [24,25] are used. The PPG recordings were collected from wearable bracelets for analysis. A total of 100 subjects participated in our experiments. Every subject was informed about the process and signed informed consents before the experiments. Subjects were asked to wear commercial bracelets while spending a whole night in a PSG testing chamber. The experimental system is shown in Figure 3. Among the 100 subjects, 4 were on a ventilator, and the data of 4 others have been severely disturbed. Therefore, the recordings of the remaining 92 subjects are used in this study.

**Figure 4:**
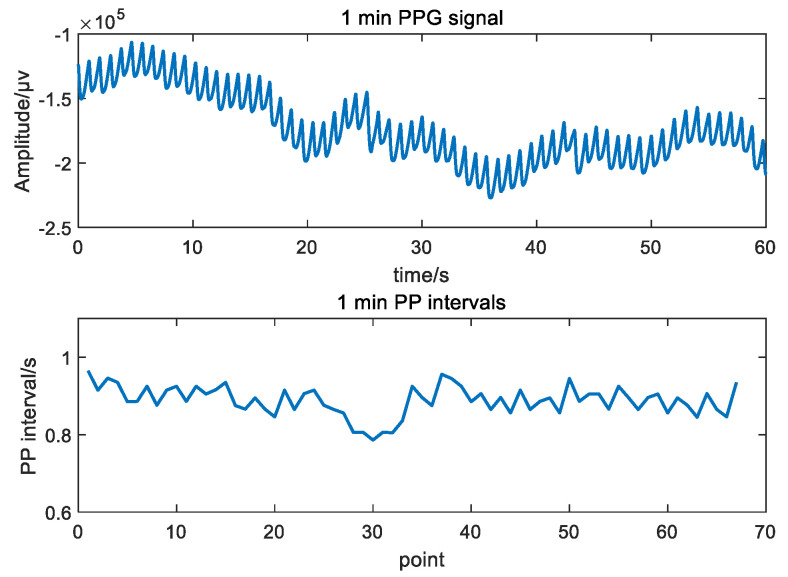
One minute PPG signal and its corresponding PP intervals.

PPG signals were preprocessed according to the following steps. First, PPG signals were segmented into 5 min epochs. Then, a local median filter was applied to these epochs to remove noise and correct signals [26]. Finally, the peaks were located with the peak detection algorithm proposed by Elgendi et al. [27]. The PP intervals (PPI) were calculated based on the located peak coordinates. Figure 4 shows an example of PPI extraction.

Registered polysomnogram technicians defined sleep stage and respiratory events for all subjects, who were divided into three groups according to the apnea–hypopnea index (AHI), namely, the average hourly number of apneic epochs [28]. Subjects with an AHI value under 5 were defined as normal (N). Those with an AHI value between 5 and 30 were defined as mild–moderate OSA (OSA-m), while above 30 was defined as severe (OSA-s). A total of 29 normal subjects, 39 OSA-m subjects and 24 OSA-s subjects were included in this study. Normal and OSA-m subjects were regarded as non-severe OSA (non-OSA-s) regarding severe OSA detection.

### 2.2. Time-Domain Indices

In the time domain, the following indices were calculated for each PPI: the mean of all PP intervals (Mean), the standard deviation of all PP intervals (SDNN), the square root of the mean of the squares of differences between adjacent PP intervals (RMSSD), the percentage of successive PP intervals that differ by more than 50 ms (PNN50) and the ratio of the standard deviation to the mean (CV) [29]. The formulas are as follows:

(1) $$Mean=\frac{1}{N}\overset{N}{\underset{i=1}{\sum }}PP_{i}$$

(2) $$SDNN=\sqrt{\frac{1}{N}\overset{N}{\underset{i=1}{\sum }}{\left(PP_{i}-\bar{PP}\right)}^{2}}$$

(3) $$RMSSD=\sqrt{\frac{1}{N-1}\overset{N-1}{\underset{i=1}{\sum }}{\left(PP_{i+1}-PP_{i}\right)}^{2}}$$

(4) $$PNN50=\frac{PP50}{TotalPP}$$

(5) $$CV=\frac{SDNN}{Mean}.$$

### 2.3. Frequency-Domain Indices

The power spectral density of the PP intervals was computed using the autoregressive Burg parametric method [30]. The power in the low-frequency band (0.04–0.15 Hz) and high-frequency band (0.15–0.4 Hz) is calculated as LF and HF, respectively [10]. LF reflects both sympathetic and parasympathetic tone while HF is driven by respiration and can estimate parasympathetic activity [31]. The LF/HF ratio is widely accepted for describing ANS balance. The formula of LF/HF is as follows:

(6) $$\frac{LF}{HF}=\frac{LF\;Power}{HF\;Power}$$

### 2.4. Information-Based Similarity of Ordinal Pattern Sequences (OP_IBS)

**Figure 5:**
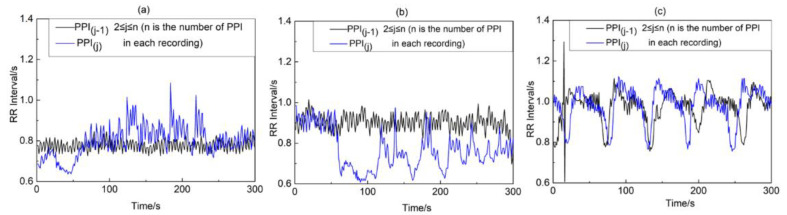
Adjacent PPI segments of (**a**) normal subject, (**b**) mild–moderate and (**c**) severe OSA subject.

**Figure 6:**
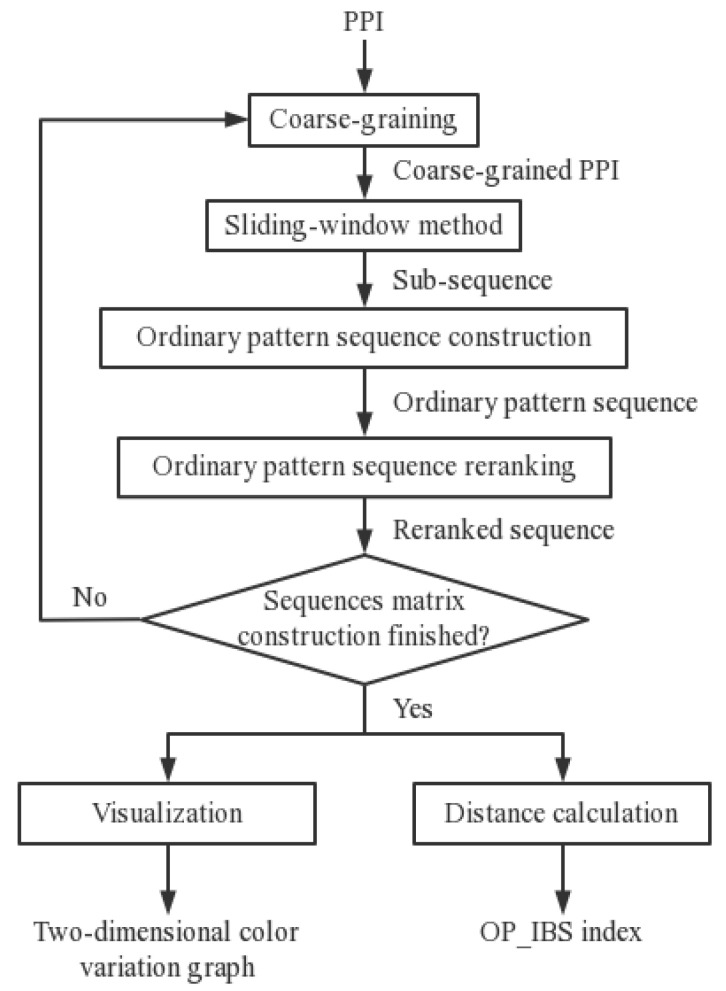
Scheme of information-based similarity of ordinal pattern sequences (OP_IBS) analysis.

Our previous study [26] has proven that the similarity between adjacent PPI segments increases as OSA becomes more severe, which is shown in Figure 5. In the OP_IBS method, the similarity between adjacent PPIs was quantified on the basis of the ordinary patterns. The scheme is shown in Figure 6. The details are shown below:

Step 1 (Coarse-graining): An n-point PPI is mean coarse-grained with a scale factor *s* as follows:

(7) $$x_{i}=\frac{1}{s}\overset{is}{\underset{j=\left(i-1\right)s+1}{\sum }}PP_{j},\;1\leq i\leq \frac{n}{s},$$

where *PP_j_* is the value of the *j*th PP interval. Coarse-graining can eliminate the interference of noise in the signal. If the scale factor is too small, the elimination effect will be diminished; if it is too large, the signal may be oversmoothed and thus important features can be lost. Values of *s* = 4–10 were tested in this study. A value of *s* = 7 turned out to be the optimal choice, which was chosen for further analysis.

Step 2 (Ordinary pattern sequence construction): For a coarse-grained PPI *X* = {$x_{1,\;},x_{2\;},\ldots \;,x_{N\;}$}, the time-delayed m-dimension series {*X*_1_, *X*_2_, …, *X_L_*} are reconstructed with a sliding window method:

(8) $$X_{L}=\left\{x_{L,\;}x_{L+\tau },\ldots ,x_{L+\left(m-1\right)\tau }\right\},\;\;L=1,2,\ldots ,N-\left(m-1\right)\tau ,$$

where *m* represents the embedding dimension, namely, the word length. *τ* represents the time delay, which equals 1 in this study.

Then, *X_L_* is reranked in ascending order:

(9) $$X_{L}^{\prime }=\left\{x_{L+\left(t_{1}-1\right)\tau ,\;}x_{L+\left(t_{2}-1\right)\tau },\ldots ,x_{L+\left(t_{m}-1\right)\tau \;}\right\},$$

where $x_{L+\left(t_{1}-1\right)\tau }\leq x_{L+\left(t_{2}-1\right)\tau ,\;}\leq \ldots \leq x_{L+\left(t_{m}-1\right)\tau }$. In the case of equal values, the elements were ordered according to the time of appearance, for instance, when $x_{a}=x_{b\;},\;x_{a\;}$ comes before $x_{b\;},$ as long as *a* < *b*. Therefore, an ordinary pattern is constructed:

(10) $$\prod_{i}=\left(t_{1},t_{2},\ldots ,t_{m}\right),\;\;i=1,2,\ldots ,m!$$

**Figure 7:**
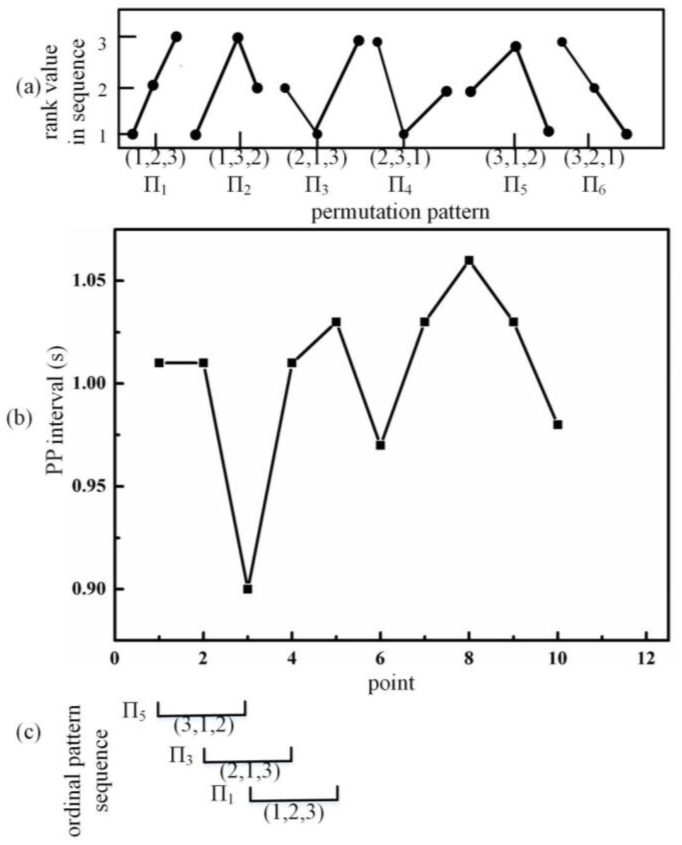
Schematic diagram for constructing an ordinal pattern sequence. (**a**) all possible ordinal patterns, (**b**) PP interval series, (**c**) corresponding ordinal patterns.

An example when *m* = 3 is presented in Figure 7. The six possibilities for Π*_i_* are listed in Figure 7a. A 10-point actual PPI signal is shown in Figure 7b. The corresponding pattern of the first three points in Figure 7b (values are 1.01, 1.01 and 0.90, respectively) is (3, 1, 2), which is Π5. The third point ranks No. 1 in the ordinal pattern because of its lowest value. As the values of the first and second points are equal, the first point ranks No. 2 because it comes first in the original PPI sequence.

Step 3 (Ordinary pattern sequence reranking): For each PPI, a series of Π*_i_* is obtained after Step 1 and Step 2. Then, the ordinal pattern series $\left\{\prod_{1},\prod_{2},\ldots ,\prod_{T}\right\}$ are reranked according to the frequencies of appearance in this PPI segment. When the numbers of occurrence are equal, the order depends on their inherent serial number as previously defined. Step 1 to Step 3 are repeated for all PPIs until an ordinary pattern sequence matrix is completed.

Step 4 (Distance calculation): The distance between an adjacent pattern sequence is calculated as the OP_IBS value of these two sequences. The equations are as follows:

(11) $$D\left(PPI_{1},PPI_{2}\right)=\frac{1}{T}\overset{T}{\underset{i=1}{\sum }}\left|r_{1}\left(\prod_{i}\right)-r_{2}\left(\prod_{i}\right)\right|W\left(\prod_{i}\right)$$

(12) $$W\left(\prod_{i}\right)=\frac{\left[-p_{1}\left(\prod_{i}\right)\log p_{1}\left(\prod_{i}\right)-p_{2}\left(\prod_{i}\right)\log p_{2}\left(\prod_{i}\right)\right]}{\sigma }$$

(13) $$\sigma =\overset{T}{\underset{i=1}{\sum }}\left[-p_{1}\left(\prod_{i}\right)\log p_{1}\left(\prod_{i}\right)-p_{2}\left(\prod_{i}\right)\log p_{2}\left(\prod_{i}\right)\right],$$

where *T* represents the total number of classes of Π*_i_*, r(Π*_i_*) is the rank order of Π*_i_*, *W*(Π*_i_*) calculates the weighting of Π*_i_* using Shannon entropy and σ is the normalization factor.

**Figure 8:**
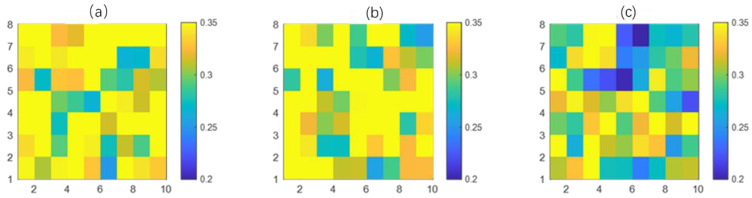
OP_IBS values for three recordings from (**a**) the normal group, (**b**) the mild–moderate OSA group and (**c**) the severe group.

Step 5 (OP_IBS calculation): For each subject, the OP_IBS values between every adjacent 5 min PPI are calculated. An example of an OP_IBS value within the recordings is shown in Figure 8. The mean value of all the OP_IBS values is obtained as the OP_IBS index for that recording.

### 2.5. Validation

Three approaches were applied to validate the calculated indices. First, correlation analyses were implemented to assess the relevance between the index and AHI value. The correlation coefficient (*R*) is a statistical measurement of the strength of a linear relationship between two variables, with a value from −1 to 1. The larger the absolute value of *R*, the stronger the linear correlation. The signs before R decide if the correlation is positive or negative. The correlation in this study is defined as follows [32]:

(14) $$\left|R\right|\in \left\{\begin{array}{cc} \left[0,\;0.2\right) & very\;weak\\ \left[0.2,0.4\right) & weak\\ \left[0.4,0.6\right) & moderate\\ \left[0.6,0.8\right) & strong\\ \left[0.8,1\right] & very\;strong, \end{array}\right.$$

Then, a significance analysis among the normal, mild–moderate OSA and severe OSA groups was carried out using a t-test and a one-way ANOVA [33]. The number of segments per class is shown in Table 1. Finally, non-OSA-s and OSA-s binary classifications were performed based on single-index and multi-index screening. The discrimination methods of machine learning were performed using the scikit-learn Python package in a 3.6.5 Python environment [34]. A decision tree classifier (number of trees = 100) [35], K-nearest neighbor (KNN, k = 5) [36], a random forest classifier [24] and Gaussian Naive Bayes [37] with default settings were employed. A 5-fold cross-validation strategy was used in the classification. The whole dataset was divided equally and randomly into five subsets. Classes were stratified on each fold. Four of them were used as the training set and the withheld set was used as the test set. Five rounds of cross-validation were performed using different partitions. Each subset was rotated as the test set while the rest were used as the training set. Accuracy (Acc), sensitivity (Sen) and specificity (Spe) represent the percentage of correctly classified samples, correctly classified OSA-s samples and correctly classified non-OSA-s samples. An F1 score was calculated to assess the classifier’s performance in the imbalanced problem [38]. The formula is as follows:

(15) $$F1\;score=2*\frac{Precision*Recall}{Precision+Recall}$$

(16) $$Precision=\frac{TP}{TP+FP}$$

(17) $$Recall=\frac{TP}{TP+FN},$$

where *TP*, *FP* and *FN* represent “true positive”, “false positive” and “false negative”, respectively. In this study, the severe OSA class was defined as positive while the non-OSA-s class was defined as negative.

### 2.6. Parameter Selection for OP_IBS

**Figure 9:**
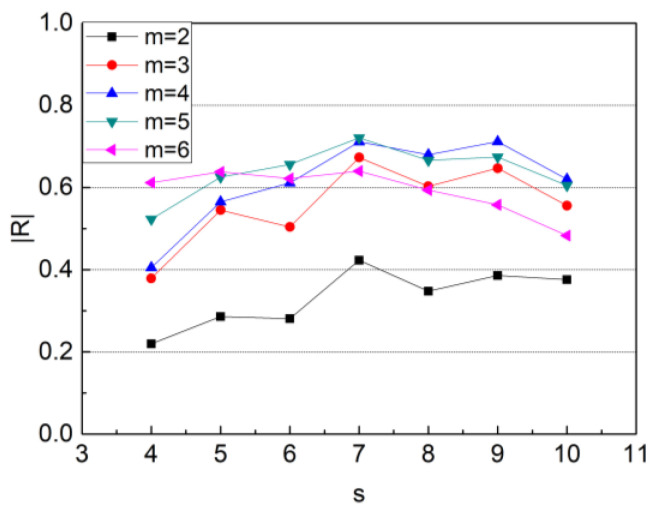
Correlation coefficient of OP_IBS with different s and m values. |R|: absolute value of correlation coefficient.

All PPI segments were coarse-grained in Step 1 by calculating the mean value of several consecutive values. Some pathological information can be highlighted after multiscale analysis [39]. Consequently, the selection of a proper scale *s* for coarse-graining was of great importance. Meanwhile, representing the range of fluctuation, the length *m* of the word in the OP_IBS calculation had a vital influence on the results. If m is too small, there are very few ordinary patterns, resulting in a large deviation in OP_IBS computing. If m is too large, some patterns will not appear due to the limited length of the data. The workload for computing will also be heavy in that case. Therefore, a heuristic was employed to find the most appropriate pair of *s* and *m* values, namely, deciding with a post hoc analysis [40]. The correlation coefficients between OP_IBS and AHI values were calculated when *s* ranged from 4 to 10 and *m* ranged from 2 to 6. The results are shown in Figure 9. When *s* = 7 and *m* = 5, the correlation reaches the highest value, which was selected for OP_IBS computing in this study.

### 2.7. OP_IBS in the Physionet Database

Though this study mainly focuses on the effectiveness of OP_IBS with wearable data, the OP_IBS method was also applied to the Physionet database [41] to verify its robustness. The database includes 70 ECG recordings from 32 subjects. Because RR extraction has a high agreement between PPG and ECG [11], it is possible to validate the OP_IBS index on that database. A total of 40 recordings are defined as the OSA group with any AHI values greater than 5 or more than 100 min apnea epochs, and 20 recordings are defined as the normal group with fewer than 5 min of breathing disorder. The OP_IBS index, along with some classical indices, was calculated on the basis of 5 min RR interval segments. Severe OSA screening was implemented. The results are compared with other research using the same database in Section 4.2.

## 3. Results

### 3.1. Similarity in Heart Fluctuation among Normal, OSA-m and OSA-s Groups

The mean ± standard deviation (SD) of time/frequency-domain indices and the OP_IBS index are listed in Table 2. The results of the IBS index in our previous work [26] are also listed for comparison. Among all indices, OP_IBS showed the highest correlation with the AHI (R = −0.721). There is also a very significant difference between the severe OSA group and the other two groups (*p* < 0.001). CV was the best-performing index in the time domain with a correlation coefficient of 0.436. Though still much lower than that of OP_IBS, it was the highest value of |R| among all linear indices. CV showed a significant difference between the OSA-s group and the normal group (*p* < 0.001) and between the OSA-s group and OSA-m group (*p* < 0.01). Frequency-domain indices performed relatively poorly in this situation. As one of the most robust indices to access ANS function [42,43], LF/HF was the only statistically significant index (*p* < 0.05 between the severe OSA group and the other two groups).

As shown in Table 2, the value of OP_IBS decreases when OSA severities increase. Because OP_IBS was able to assess the similarity of two series, the results show that the similarity between adjacent PPI segments increases as OSA becomes more severe. This finding is consistent with our IBS study [26].

### 3.2. Severe OSA Screening

As shown in Table 2, significant differences only exist between the OSA-s group and the other two groups. A decision tree classifier, KNN, a random forest classifier and Gaussian Naive Bayes were applied to implement severe OSA screening based on OP-IBS. The results are presented in Table 3. The decision tree classifier achieved a relatively high accuracy (85.9%) while maintaining a good balance between sensitivity (79.2%) and specificity (88.2%). Thereby, the decision tree classifier was selected for further analysis.

While OP _IBS performed the best in the significance analysis, IBS, CV and LF/HF also performed relatively well. IBS is an index, the effectiveness of which was proved in our previous work [24]. CV and LF/HF are the classical indices in the time and frequency domains, respectively. Single-index and multi-index screening were implemented on the basis of these indices employing the decision tree classifier. The results are presented in Table 4. Both CV (*p* < 0.001), IBS (*p* < 0.001) and OP_IBS (*p* < 0.001) show a significant difference between the OSA-s and non-OSA-s groups, while there is no significant difference in LF/HF (*p* = 0.079) between these two groups. In the single-index screening, OP_IBS performed the best, with 85.9% accuracy, 79.2% sensitivity and 88.2% specificity. IBS also has a good performance with 81.5% accuracy. CV and LF/HF only achieve an accuracy of 68.5% and 70.7%, respectively. The highest F1 score (74.5%) of OP_IBS also represents a good balance between sensitivity and specificity. In the multi-index screening, the combination of all indices performed best, with an accuracy, sensitivity, and specificity all above 90%. The results show that OP_IBS is a robust index in severe OSA screening and can effectively improve the classical HRV analytical methods.

## 4. Discussion

### 4.1. Comparison with Studies Using Wearable Data

With the increasing attention paid to daily healthcare and the growing popularity of wearable devices, an increasing number of studies have focused on detecting OSA using off-the-shelf portable devices. Scientists turned their eyes to traditional ECG signals first. However, the strict measuring requirements make ECG devices not suitable for long-time monitoring. Wearable ECG devices require sticky metal electrodes and conductive gel, causing uncomfortableness for subjects. As a result, the sensors can be easily displaced and thus cause low detection accuracy [44]. As presented in Table 5, the accuracy is relatively low with wearable ECG devices. The accuracies are all below 80% whether in a single index or multi-index screening [45,46].

By contrast, PPG devices are more applicable in real life. As optical devices, PPG devices can detect blood volume changes through a light source and a photodetector on the surface of the skin [15]. This makes PPG measurement more acceptable. Less displacement during wear improves detection accuracy. Overall, the accuracy with PPG was relatively higher. In moderate–severe OSA detection, Hayano et al. analyzed PPG data and achieved a good accuracy of 85% [47]. However, the limited database (only 41 subjects) may be a noteworthy drawback. Papini et al. conducted research on a large number of samples, extracting 212 indices and putting them into the convolutional neural network for screening [48]. They achieved good performance in predicting AHI and achieved an accuracy of 91.3% in severe OSA screening. However, the imbalance problem between sensitivity and specificity also needs attention.

In our proposed method, we found that OP_IBS is a robust index to detect severe OSA patients. With a decision tree classifier, an accuracy of 85.9% was achieved in a single-index screening. As shown in Table 3, there also exists a good balance between sensitivity (79.2%) and specificity (88.2%). The combination of OP_IBS, CV, LF/HF and IBS performed better in this situation. It reached 91.3% accuracy, 91.0% sensitivity and 91.5% specificity. Compared with our former study based on the same database [24], this new OP_IBS method significantly improves the screening accuracy.

### 4.2. Comparison with Studies on the Physionet Database

The OP_IBS method was applied to the Physionet database [41] and compared with other methods to adopt a more comprehensive analysis. The comparison with previous studies using the same database is listed in Table 6 [10,49,50]. The classification boundary was set to 5. On the one hand, it can facilitate comparison. On the other hand, the significance of detecting OSA in the early stage was also taken into consideration. Meanwhile, because the quality of data in the Physionet database is better than that from commercial bracelets, even the ANS disorders caused by mild OSA could be detected. As shown in Table 6, OP_IBS performed well. It achieved the highest accuracy of 91.7%. The sensitivity (95%) and specificity (85%) were also relatively high with a good balance. These results prove the robustness and applicability of the IBS method.

### 4.3. OP_IBS Method and Parameter Selection

An appropriate length of the segment is crucial in OSA analysis. In too short a segment, the apnea events can be easily distorted [51]. When the segment is too short, an apnea reaching the threshold of 10 s can be divided into 2 epochs and thus misdetected. A 5 min series is indicated to be the standard length for heart rate variability [52,53]. The segmentation rule of 5 min is common and effective in OSA detection [4,54].

Previous studies have proven the correlations of heartbeat dynamics with heart rate time series [55]. This correlation is influenced by physical decline and diseases [55,56]. However, present studies have rarely quantified such a correlation. Heart rate is highly controlled by the autonomic nervous system, which is nonlinear and dynamic [17]. Moreover, the apnea regulation of HR was also not linear [57]. As a result, nonlinear analysis methods are demanded in this circumstance. Able to quantify the similarity between two symbolic sequences, the IBS index is proposed as a quantitative index in heart rate assessment [26,58].

In this study, OP_IBS is proposed for OSA detection. First, the coarse-graining process captured the information at multiple temporal scales [4]. The screening performance was improved during a search for the best parameters. Previous work has reflected the advantages of using various scales in HRV research [59]. In addition, IBS was proven to be superior in nonlinear physiological information analysis. In Wu et al.’s work, IBS was employed in OSA assessment [26]. The change in HR was analyzed without being affected by the amplitude and absolute proportion of the specific pattern appearance. However, the binarization of the PP series is highly dependent upon the relationship between adjacent PP values. Some large-scale characteristics can be neglected in this way. Therefore, OP_IBS was introduced. In this case, an OP was constructed based on the order of consecutive values. Same-length series can generate more possible permutation patterns now. For example, the total number of possibilities of permutation patterns for a 5-point sequence is 5! (120) right now, far more than 2^D^ (32) in the IBS method. More patterns were taken into consideration, and thus, the change in HR dynamics is better reflected.

Parameter selection was implemented to enhance the performance of OP_IBS. Coarse-graining is a common way to highlight pathological information and eliminate noise [39,60]. Values of s = 4–10 were tried in this process. Determining the length of words and the total number of possibilities of patterns, m played a vital role in OP_IBS calculation. Too short a word leads to significant deviation, while too long a word leads to a redundant computation. Therefore, OP_IBS was calculated when m = 2–6. The correlations between AHI and OP_IBS are presented in Figure 9. The index showed the best performance with s = 7 and m = 5, which were chosen in subsequent calculations.

### 4.4. Physiological Significance

Capable of analyzing HR dynamics and calculating the similarity, OP_IBS was proposed based on the classical IBS method. While retaining the advantages of assessing HR fluctuation regularity, it considers more possibilities and can analyze more subtle differences among epochs. Therefore, OP_IBS can capture the regularity of HR nonlinear dynamics caused by OSA more comprehensively, making it more suitable for OSA assessment and classification.

In the present study, the significant difference of LF/HF and OP_IBS only happened between OSA-s and the other two groups (Table 2). The significant difference only occurred in the severe OSA group, probably because of the low quality of the data. Lacking strict conditions, the process of collecting data from wearables can be easily disturbed [15]. Because ANS dysfunction worsens with the deterioration of the disease, HR changes in mild–moderate OSA subjects may be too negligible to be detected. Blomster et al. argued that mild OSA would not modulate baroreflex sensitivity, which is a possible representation of impaired cardiac autonomic control [61]. Patients with severe OSA may suffer from more frequent apnea than those with mild or moderate OSA [62]. Insufficient oxygen saturation may stimulate sympathetic nerve activity directly [63], thus leading to a more severe disorder. In contrast, OP_IBS performed well in early-stage OSA detection on the Physionet database. The data in the Physionet database are of better quality due to being collected from an experimental environment. For OSA screening in the early stage, OP_IBS shows a significant difference between the two groups and can obtain a good accuracy (91.7%).

LF/HF is proven to be one of the most robust indices to access ANS balance [64]. The significant difference of LF/HF between OSA-s and the other two groups is consistent with previous studies [10]. This finding verifies the ANS imbalance in the OSA-s group. OP_IBS is proposed to assess the similarity between time series. The decreased value of OP_IBS in the OSA-s group proves HR dynamic changes. OSA patients were proven to have increased sympathetic tone and decreased parasympathetic activity [65]. As the disease deteriorates, parasympathetic activity is increasingly inhibited. The parasympathetic control of heart rate is one of the main reasons for the patterns of bradycardia and tachycardia during apnea [66]. This may cause the increased similarity of adjacent PPIs in the OSA-s group.

## 5. Conclusions

This study proposes the OP_IBS method to assess the similarity between adjacent PPIs using wearable bracelets. The results show that the accuracy of OP_IBS in severe OSA detection is 85.9%, much better than classical LF/HF (70.7% accuracy). When combined with some other effective indices (CV, LF/HF and IBS), a good performance with 91.3% accuracy, 91.0% sensitivity and 91.5% specificity was achieved. Compared with other studies on wearable devices, our method shows superior screening capabilities [45,46,47,48]. OP_IBS also has a good robustness. In the Physionet database, OP_IBS performed exceptionally well in early screening with an accuracy of 91.7%. Its performance is better than most peer studies [10,49,50]. Therefore, OP_IBS provides a new perspective into HR dynamics in OSA analysis and could be utilized in OSA screening.

## Figures and Tables

**Table 1:** Number of 5 min segments in N, OSA-m and OSA-s groups.

	N (29)	OSA-m (39)	OSA-s (24)
Mean ± SD	80.86 ± 11.54	83.49 ± 15.12	84.03 ± 12.78
Total number	2345	3256	2018

N: normal group; OSA-m: mild–moderate OSA group; OSA-s: severe OSA group; SD: standard deviation.

**Table 2:** Indices for N, M and S groups.

Indices		Correlation		N	OSA-m	OSA-s	*p*-Value		
		R	*P*				N&OSA-m	N&OSA-s	OSA-m&OSA-s
Time domain	Mean	−0.254	0.015	0.962 ± 0.116	0.951 ± 0.101	0.907 ± 0.087	0.674	0.058	0.105
	SDNN	0.327	0.001	0.072 ± 0.024	0.076 ± 0.020	0.089 ± 0.026	0.507	0.008	0.027
	RMSSD	0.180	0.086	0.066 ± 0.022	0.071 ± 0.022	0.077 ± 0.026	0.351	0.100	0.381
	PNN50	0.123	0.242	25.558 ± 15.474	26.572 ± 16.397	29.336 ± 15.624	0.796	0.392	0.505
	CV	0.436	0.000	0.076 ± 0.023	0.081 ± 0.020	0.099 ± 0.028	0.402	<0.001	0.003
Frequency domain	LF	0.268	0.010	0.002 ± 0.001	0.002 ± 0.001	0.003 ± 0.002	0.973	0.085	0.072
	HF	0.112	0.288	0.0012 ± 0.0011	0.0012 ± 0.0008	0.0013 ± 0.0010	0.870	0.602	0.689
	LF/HF	0.255	0.014	2.356 ± 0.932	2.285 ± 0.838	3.052 ± 1.843	0.812	0.039	0.016
Nonlinear	IBS	−0.653	0.000	0.297 ± 0.016	0.295 ± 0.012	0.266 ± 0.028	0.634	<0.001	<0.001
	OP_IBS	−0.721	0.000	0.351 ± 0.021	0.348 ± 0.014	0.300 ± 0.040	0.705	<0.001	<0.001

Mean: The mean of all PP intervals; RMSSD: the square root of the mean of the squares of differences between adjacent PP intervals; PNN50: the percentage of adjacent PP intervals greater than 50 ms; LF: low-frequency power; HF: high-frequency power; LF/HF: the ratio of low-frequency power to high-frequency power; OP_IBS: information-based similarity of ordinal pattern sequence; N: normal group; OSA-m: mild–moderate OSA group; OSA-s: severe OSA group; R: correlation coefficient; *p*-value: significance of difference.

**Table 3:** Comparison of severe OSA screening results with different classifiers based on OP_IBS.

	Acc/%	Sen/%	Spe/%	F1 Score/%
Decision tree	85.9	79.2	88.2	74.5
Random Forest	83.7	73.0	88.2	69.7
K Nearest Neighbor	83.6	70.0	89.5	67.9
Naive Bayes	86.8	51.8	97.1	62.5

Acc: accuracy; Sen: sensitivity; Spe: specificity.

**Table 4:** Comparison between screening results of non-OSA-s and OSA-s groups.

Indices	*p*-Value	Acc/%	Sen/%	Spe/%	F1 Score/%
CV	0.000	68.5	37.5	79.4	38.3
LF/HF	0.079	70.7	54.2	76.5	49.1
IBS	0.000	81.5	66.3	86.2	66.2
OP_IBS	0.000	85.9	79.2	88.2	74.5
OP_IBS, IBS	\	86.8	72.7	91.0	74.2
OP_IBS, IBS, LF/HF, CV	\	91.3	91.0	91.5	81.3

non-OSA-s: non-severe SA group; OSA-s: severe SA group; Acc: accuracy; Sen: sensitivity; Spe: specificity.

**Table 5:** Comparison between proposed method and previous studies regarding wearable data.

Device	Signal	Subject	Feature	Classifier	Criterion	Result		
						Acc/%	Sen/%	Spe/%
Single-lead ECG patch [45]	ECG	119	single feature (CVHR)	ROC curve, Youden index	AHI = 15	64.7 *	52.9	94.1
Wearable ECG-Belt [46]	ECG	241	multiple features (11 features)	SVM	AHI = 15	72	70	74
Wearable watch device [47]	PPG	41	single feature (Fcv)	ROC curve	AHI = 15	85	82	89
Wrist-worn rPPG [48]	PPG	188	multiple features (212 features)	CNN	AHI = 30	91 *	46	98
Wearable bracelet [24]	PPG	92	multiple features (sIBS, dIBS, STD, LF.)	Random Forest	AHI = 30	84.7	76.7	89.6
Wearable bracelet #	PPG	92	single feature (OP_IBS)	Decision Tree	AHI = 30	85.9	79.2	88.2
Wearable bracelet #			multiple features (CV, LF/HF, IBS, OP_IBS)			91.3	91.0	91.5

#: Our proposed method. *: The result was inferred according to the paper; CVHR: Cyclic variation of heart rate; Fcv: Hourly frequency of cyclic variation of heart rate; OP_IBS: information-based similarity of ordinal pattern sequence; ROC: receiver operating characteristic; SVM: support vector machine; CNN: convolutional neural network; Acc: accuracy; Sen: sensitivity; Spe: specificity. STD: the standard deviation of all PP intervals.

**Table 6:** Comparison of classification results between the proposed method and previous studies in Physionet database.

Reference	Feature	Number of Recordings	Length of RR Segment	Classification Boundary	Classification Results
Liu et al. [49]	The Hilbert–Huang transform (HHT) based cardiopulmonary coupling	69	1 min	5	Acc = 79.1% Sen = 73.1% Spe = 71.2%
Li et al. [10]	Sliding trend fuzzy approximate entropy	60	5 min	5	Acc = 85.0% Sen = 82.5% Spe = 90.0%
Pietrzak et al. [50]	Standard deviation of successive differences	70	10,000 s	5	Acc = 88.5% Sen = 96.0% Spe = 70.0%
Our proposed method	Information-based similarity of ordinal pattern sequences	60	5 min	5	Acc = 91.7% Sen = 95.0% Spe = 85.0%

## Data Availability

Not applicable.
